# Is faster economic growth compatible with reductions in carbon emissions? The role of diminished population growth

**DOI:** 10.1088/1748-9326/12/1/014003

**Published:** 2017-01-05

**Authors:** Gregory Casey, Oded Galor

**Affiliations:** 1 Department of Economics, Brown University, 64 Waterman Street, Providence, RI 02912, USA; 2 Institute at Brown for Environment and Society, Brown University, 85 Waterman Street, Providence, RI 02912, USA; 3 Population Training and Studies Center, Brown University, 68 Waterman Street, Providence, RI 02912, USA

**Keywords:** climate change, economics, demography

## Abstract

We provide evidence that lower fertility can simultaneously increase income per capita and lower carbon emissions, eliminating a trade-off central to most policies aimed at slowing global climate change. We estimate the effect of lower fertility on carbon emissions, accounting for the fact that changes in fertility patterns affect carbon emissions through three channels: total population, the age structure of the population, and economic output. Our analysis proceeds in two steps. First, we estimate the elasticity of carbon emissions with respect to population and income per capita in an unbalanced yearly panel of cross-country data from 1950–2010. We demonstrate that the elasticity with respect to population is nearly seven times larger than the elasticity with respect to income per capita and that this difference is statistically significant. Thus, the regression results imply that 1% slower population growth could be accompanied by an increase in income per capita of nearly 7% while still lowering carbon emissions. In the second part of our analysis, we use a recently constructed economic-demographic model of Nigeria to estimate the effect of lower fertility on carbon emissions, accounting for the impacts of fertility on population growth, population age structure, and income per capita. We find that by 2100 C.E. moving from the medium to the low variant of the UN fertility projection leads to 35% lower yearly emissions and 15% higher income per capita. These results suggest that population policies could be part of the approach to combating global climate change.

## 1. Introduction

Population growth is a major driver of carbon emissions, both historically and in projections of future emissions [1, 2]. Yet, relatively little attention has been devoted to investigating the potential for population policies to influence global climate change [3]. Motivated by this fact, this paper examines the effect of lower fertility on carbon emissions, taking into account three crucial channels: total population, the age structure of the population, and output per capita. We provide evidence that lower fertility can simultaneously increase income per capita and lower carbon emissions, even without taking into account economic damages from climate change. This result stands in stark contrast to other environmental policies, such as carbon taxes and cap-and-trade policies, which must balance environmental benefits against lost output [4]. Thus, our results suggest that population policies could serve as an effective tool to combat global climate change, while sustaining economic growth.

Our analysis proceeds in two steps. First, we estimate the partial elasticities of carbon emissions with respect to population, output per person, and the age structure of the population [5, 6]. Consistent with existing literature, we find that the partial elasticity of emissions with respect to population is larger than the elasticity with respect to output per person [7], and we are the first to provide formal statistical evidence for this fact. The partial elasticity of emissions with respect to population is nearly 7 times greater than the elasticity with respect to income per capita. This implies that 1% slower population growth could be accompanied by an increase in income per capita of nearly 7% while still decreasing carbon emissions, eliminating a trade-off central to other environmental policies.

By themselves, these STIRPAT regressions are insufficient to measure the total impact of changes in population on emissions, because population growth will affect carbon emissions both directly and through the other explanatory variables [3, 8]. Hence, the second step of our analysis employs a recently developed economic-demographic model of Nigeria to estimate the effect of lower fertility on both carbon emissions and income per capita [9]. The model was developed to estimate the effect of fertility on income per capita, and we use our regression results to estimate the impact of lower fertility on emissions. We find that by 2100 C.E. moving from the medium to the low variant of the UN fertility projection leads to 35% lower yearly emissions and 15% higher income per capita.

These results have important implications for climate change policy. It is widely accepted, and enshrined in international agreements, that the burden of mitigating global climate change needs to vary between rich and poor countries in order to ensure that developing countries can continue to experience economic growth and poverty reduction [10, 11]. At the same time, the projected economic and population growth in the developing world indicates that these poorer countries will be substantial contributors to climate change [12]. Thus, policy options that will lessen emissions from developing countries without impeding economic development appear desirable. Our analysis suggests that population policies could achieve this difficult goal. Moreover, since population policies could eliminate the trade-off between environmental and economic priorities, they may not suffer from the free-rider problems that pose a central challenge in current approaches to mitigating global climate change [13,14].

To the best of our knowledge, we are the first to demonstrate how population policies can simultaneously increase income per capita and lower carbon emissions, eliminating a trade-off central to existing policy proposals. Our paper, however, is closely related to two existing literatures. The first is the literature estimating the STIRPAT equation [7, 15]. Our key contribution to this literature is to examine how the STIRPAT equation provides evidence for the ability of reductions in population growth to achieve both economic and environmental priorities. From a statistical perspective, we build on the existing STIRPAT literature by formally testing the difference in coefficients between population and income per capita and by using an updated dataset for output per person. Second, our work is related to applications of the population-energy-technology (PET) model that estimate the effect of exogenous changes in population and urbanization on carbon emissions [2,3,16]. We build on this literature by expressly examining economic outcomes and by considering a broader range of channels through which changes in fertility affects these economic outcomes.

## 2. The STIRPAT equation

### 2.1 Methods

The first step of our analysis is to estimate the elasticity of carbon emissions with respect to income per capita and population. To do so, we estimate the STIRPAT equation:

(1) $$I_{i,t}=P_{i,t}^{a}A_{i,t}^{b}T_{i,t}^{\tilde{c}}e_{i,t},$$

where *I*_*i,t*_ is environmental impact in country *i* at time t, *P* is population, *A* is affluence (income per capita), *T* is technology, and e is the residual error term. A substantial literature analyzes STIRPAT regressions to examine the determinants of many measures of the environmental impact of human activity [7]. We focus on total carbon emissions.

The STIRPAT equation is derived from the IPAT accounting identity [17, 18], and most applications of STIRPAT are focused on decomposing environmental impacts between explanatory variables. This decomposition can be aimed at explaining past emissions or predicting future emissions. Our goal is different. We want to understand the effect of changes in fertility on both environmental and economic outcomes, accounting for the effect of fertility on population levels, population age structure, and income per capita. Thus, we use the partial elasticities from the regression equation to parameterize the economic-demographic model (see section 3).

Given our goal, the difference between the coefficients on population and affluence is of primary importance. Thus, in all regressions, we test the null hypothesis that these coefficients are equal (i.e., *a* = *b*). While the literature provides a wide range of estimates for both coefficients—depending on the dependent variable under consideration and the choice of regression specification—we are the first to test for a difference in coefficients between population and affluence [7]. If the coefficient on population is significantly larger than the coefficient on income per capita, then decreases in population could potentially lower carbon emissions even while substantially increasing income per capita, overcoming the trade-off central to most environmental policies.

To estimate equation (1), it is necessary to assume a specification for technology (T). We make the following assumption:

(2) $$\ln T_{i,t}={\tilde{f}}_{i}+{\tilde{g}}_{t}+h\ln S_{i,t}+x_{i,t}^{\prime }\tilde{\delta },$$

where ${\tilde{f}}_{i}$ is a fixed effect capturing time-invariant differences between countries, ${\tilde{g}}_{t}$ is a fixed effect capturing differences in global technology over time that affect all countries, S_i,t_ is a measure of the age structure of the population, and *x*_*i,t*_ is a set of control variables including urbanization and trade. All three of the time-varying explanatory variables have been found to affect carbon emissions in the existing literature [2, 15, 19]. The inclusion of age structure, *S*_*i,t*_, is important for our results since changes in fertility patterns mechanically alter the age structure of the population, implying that we need to capture this effect in the economic-demographic model. In the appendix (available at stacks.iop.org/ERL/12/014003), we also include income per capita squared to capture the environmental Kuznets curve (EKC), but the term is insignificant in our main specification.

Recent advances in the STIRPAT literature have demonstrated the importance of correcting for potentially non-stationary variables [15,20]. Thus, our main specification estimates a log-linearized version of (1) in first differences. Thus, our estimating equation becomes:

(3) $$\ln I_{i,t}-\ln I_{i,t-1}=a\left(\ln P_{i,t}-\ln P_{i,t-1}\right)\;\;+b\left(\ln A_{i,t}-\ln A_{i,t-1}\right)\;\;+c\left(\ln S_{i,t}-\ln S_{i,t-1}\right)+{\left(x_{i,t}-x_{i,t-1}\right)}^{\prime }\delta \;\;+\left(g_{t}-g_{t-1}\right)+\left(\ln e_{i,t}-\ln e_{i,t-1}\right),$$

where $c=\tilde{c}h$, $\delta =\tilde{\delta }\tilde{c}$, and $g_{t}=\tilde{c}{\tilde{g}}_{t}\;\forall t$. It is important to note that the coefficients on population and affluence are still the same as in equation (1).

Our equation is estimated on an unbalanced yearly panel of countries. We use standard sources for all data. Our dependent variable is carbon emissions from production, which are from Oak Ridge National Laboratory [21]. Our measures of population and income per capita come from the Penn World Tables (PWT) version 8.0 [22]. We employ the newly created output-side measure of income per capita, which is the best match for our emissions measure. Age structure, urbanization and trade data are all from the World Bank’s World Development Indicators (WDI) database. To capture age structure, we use the fraction of population of between the ages of 15–64, which we denote as ‘working age’. In the appendix, we show that all our results are robust to alternate measures of income, alternate samples, and alternate estimation strategies. Summary statistics are provided in appendix table A1.

### 2.2 Results

**Figure 1.**
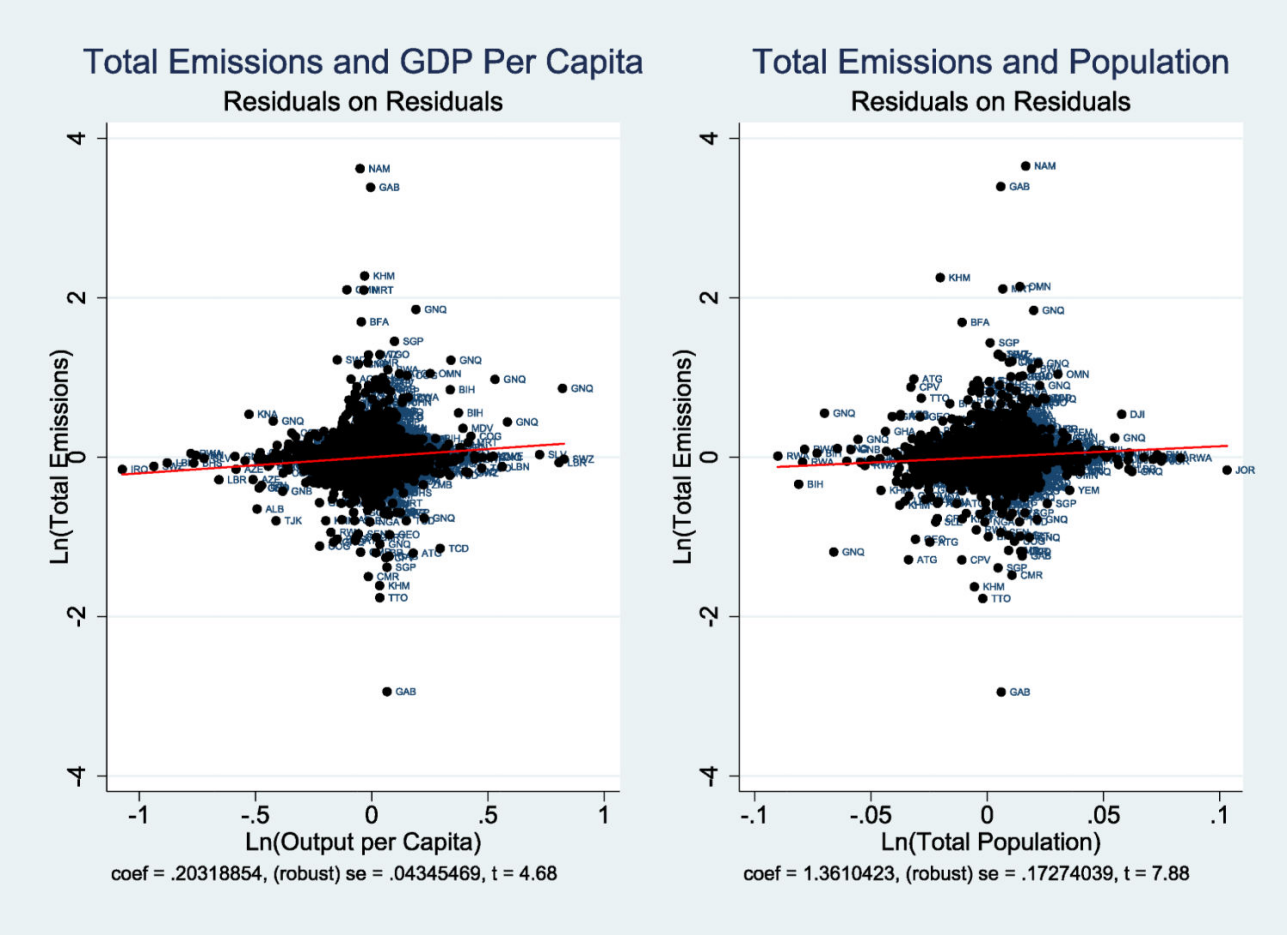
Partial residual plot from column 1 in table 1. Visual inspection of the role of outliers requires a difference in the x-axis between the two panels, which obscures the fact that the coefficient on population is larger.

Table 1 presents the results of the regression using equation (3). In column 1, we present a simple regression with only population and income per capita as explanatory variables. This specification highlights the potential for lower population to decrease emissions and increase income per capita simultaneously. Specifically, the coefficient on income per capita is 0.203, while the coefficient on population size is 1.364, a 6.7-fold difference. The difference is statistically significant at the 0.1% level. The difference in coefficients implies that a decrease in population could potentially decrease emissions and raise income per capita as long as the elasticity of income per capita with respect to population is less than 6.7. Thus, decreases in population growth could mitigate environmental concerns while permitting further economic growth. To ensure that this result is not driven by outliers, figure 1 presents the residual scatter plots from the regression in column 1.

Column 2 adds the share of the working age population, the other key variable to be affected by a change in fertility. While statistically significant, the inclusion of the working age population has little effect on the population and income per capita coefficients. To ensure that our results are not driven by omitted variables, the final two columns add controls for urbanization and trade. Again, the key results are unchanged. In all cases, the equality of coefficients can be rejected at the 0.1% level. Importantly, the regression coefficients are not substantially altered by the inclusion of urbanization or trade. If urbanization was an important channel through which population led to increases in emissions, the coefficient on population would likely have decreased substantially once urbanization was included as a control variable in the regression. Our preferred specification is column 4, which includes controls for the major confounding variables identified in the literature. Thus, we use this specification to parameterize the economic-demographic model in the second phase of our analysis.

In the appendix, we show that our key qualitative result—the large difference in coefficients between population and income per capita—holds in a number of other settings. To ensure that the results are not driven by attenuation bias, which can be exacerbated by differencing, we demonstrate that the results hold when estimating the equation in levels (table A2). In this case, the squared term on income per capita becomes significant. While our goal is not to provide a detailed examination of the EKC relationship, the fact that first differencing removes the significance of the squared term is consistent with existing literature [23]. We also show that the results are unaffected by moving to a balanced sample of countries, indicating that the results are not driven by the changing sample (table A3). We also re-estimate the STIRPAT equation using total income, instead of income per capita (table A4). The population coefficient is statistically significant in this specification, further supporting the finding that population matters beyond simply increasing total output. Finally, we show that the qualitative results are unchanged if we use several other measures of income per capita. We use the consumption-side (table A5) and national accounts (table A6) measures from the PWT, indicating that our results are not driven by the use of the output-side measure. We also use the exchange-rate based measure from the WDI (table A7), demonstrating that our results are not a byproduct of the adjustments for price differences across countries.

### 2.3 Discussion of regression results

The regression coefficients presented in table 1 capture the effect of the explanatory variables on carbon emissions through two key channels, the energy intensity of output and the carbon intensity of energy, in addition to their direct effects. Unfortunately, regressions of this type cannot tell us more about the specific mechanism through which population and output affect carbon emissions. Since the employed economic-demographic model does not explicitly capture the energy intensity of output or the carbon intensity of energy, we rely on the simplified reduced- form relationship provided by the STIRPAT regression to parameterize the effects of population, age structure, and income per capita on carbon emissions. Understanding the exact causal mechanisms underlying these regression results is an interesting and important way forward for future work in this area.

While we are the first to formally test for the difference in coefficients between population and income per capita, these results are consistent with the existing literature. Jorgensen and Clark estimate an equation similar to ours, and the results display the same qualitative pattern [19]. Specifically, they find a population elasticity of 1.43 and an income per capita elasticity of 0.65 in their first-differenced specification with similar results in alternate specifications. Our major differences in specification, in addition to formally testing for different coefficients, include the use of new data, differing time scales, the inclusion of age structure, and the use of time fixed-effects in all specifications. They also find that the elasticities are relatively stable across time and space [19, 24]. Knight *et al* also find similar results when focusing on alternate population measures such as employed persons and hours worked [25]. For example, when also controlling for hours worked, they find a population elasticity of 2.25 and an income per capita elasticity of 0.59. Earlier work, which did not use panel data to mitigate omitted variable bias, finds similar coefficients for population and income per capita [5, 6]. More exhaustive reviews of elasticities found in the existing literature, as well as discussions of different estimation techniques and specifications, can be found in existing work [2,7,15].

More recently, a growing literature has included intensity variables, such as the energy intensity of output, in STIRPAT regressions and found more similar coefficients between population and income per capita [7, 20, 26]. This addition is an important step forward in accounting applications of STIRPAT, but is not appropriate for our purpose. Our goal is to determine whether decreases in fertility can simultaneously achieve economic and environmental policy priorities. As noted above, the economic-demographic model does not have an explicit energy sector, and therefore, the appropriate regression coefficients must include the effect of population and income on carbon emissions via the energy intensity of output and the carbon intensity of energy. Earlier results including intensity variables suggest that the difference in elasticities between population and income per capita could be explained by a greater effect of population on the energy intensity of output, which is an interesting area for further study. As discussed in section 3.2, the careful modeling of fertility and omission of an explicit energy sector in the economic-demographic model represent a trade-off when compared to modeling strategies based on PET [2, 3, 16]. Section 4 discusses several ways that the current analysis could be extended in future work, including more explicit modeling of the energy sector.

## 3. The impact of fertility on economic and environmental outcomes

### 3.1. Methods

The second step of our analysis quantifies the effect of lower fertility on economic and environmental outcomes. STIRPAT regressions, while useful for decomposition exercises, are insufficient for determining the overall environmental impact of an exogenous change in an explanatory variable [3,8]. The regression cannot tell us about the relationship between the explanatory variables. To fully account for these interdependencies, we use the economic-demographic model developed by Ashraf, Weil, and Wilde (AWW) [9]. The model was constructed explicitly to evaluate the effect of changes in fertility on income per capita, making it well-suited for our purposes. We examine the effect of an exogenous reduction in fertility on both economic and environmental outcomes in Nigeria. As in the original analysis, our exogenous change in fertility is a movement from the medium to the low variant of the UN fertility projections. We use the most recent projections [27].

The AWW model examines the effect of fertility on economic growth through several channels, which can be divided into three main categories. We call the first category *composition effects.* Changes in fertility alter the age structure of the population, which affects economic output through the number of people of working age (the ‘dependency effect’), savings behavior (the ‘life-cycle saving effect’), and labor supply differences within the working age population (the ‘life-cycle labor supply effect’). We deem the second category *behavioral effects,* which encompasses changes in economic behavior for an individual as a direct result of having children. When fertility is reduced, parents have more time to work (the ‘childcare effect’) and can invest more resources in the education of each child (the ‘child-quality effect’). The third category is *factor accumulation.* High fertility reduces the amount of physical capital per person (the ‘Solow effect’) and natural capital per person (the ‘Malthus effect’). Moreover, the increase in labor force participation caused by lower fertility leads to greater human capital via work experience (the ‘experience effect’).

We use the AWW model to measure the effect of the change in fertility on the total population level, the age structure of the population, and income per capita. We then combine the model output with our regression results from column 4 in table 1 to estimate the impact on carbon emissions. Since we do not know the future values for the time fixed effects, we estimate the ratio of carbon emissions between the two scenarios.

Our work is closely related to analyses that estimate the effect of exogenous changes in population and urbanization on carbon emissions using the PET model [2, 3, 16]. The key difference between the analyses is that the present paper is expressly interested in the effect of fertility on both economic and environmental outcomes. Thus, we use an economic-demographic model specifically designed to estimate the effects of fertility on economic growth, accounting for all of the channels discussed above. The earlier works focus on composition effects and do not report economic outcomes.

This approach involves trade-offs. The PET model captures rich details of the population composition and energy sector, but only examines some of the channels through which fertility affects economic outcomes. Another strength of the AWW model lies in the careful selection of well identified parameters taken from the existing microeconomic literature. Thus, the parameters are strongly grounded in the historical experience of Nigeria. The strict requirements for parameterizing the model, however, imply that it can only be applied in a single country, unlike the PET model. Also, the demographic model does not explicitly model the energy sector. Instead, we use the STIRPAT regressions to capture the reduced-form effects of population, age structure, and income per capita on carbon emissions.

### 3.2. Results

**Figure 2.**
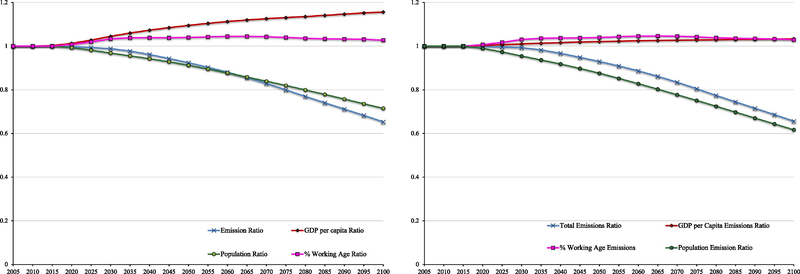
Results from the economic-demographic model. All variables are the ratio of the outcome of the low fertility scenario over the medium fertility scenario. *Panel A* (left) plots the main outcome variables. *Panel B* (right) decomposes the difference in emissions between sources.

The results of our analysis are presented in figure 2. In all cases, results are presented as the ratio of the outcome under the low fertility scenario compared to the outcome under the medium fertility scenario. Panel A presents the outcomes of the major variables in the analysis. Emissions are sharply lower under the low fertility scenario, while income per capita increases. This is the key qualitative message of our analysis. Specifically, relative emissions are 10% lower by 2055 and 35% by 2100. Income per capita, meanwhile, is 10% higher in 2055 and 15% in 2100. Thus, the income gains occur sooner, while emission reductions are back-loaded.

The share of the population that is of working age increases slightly as a result of the change in fertility patterns. At its highest point, the share is 4.5% higher than it would have been under the medium fertility scenario. Relative population follows a path very similar to that of total emissions, demonstrating how strongly changes in population levels drive emissions.

Panel B translates these effects into their impact on emissions. As suggested by panel A, emission reductions due to lower population drive the results. Increases in the working age fraction of the population and income per capita have only small positive effects on relative emission levels. Between the two, the change in the working age share has a bigger effect on emissions than does the increase in income per capita, though the effects become more similar over time.

The appendix includes results when using alternate specifications and measures of income per capita (figures A3–A5). In all cases, the qualitative effects are similar. The most significant difference occurs when using the balanced regression sample (figure A2) or estimating the regression in levels (figure A1). In these cases, relative emissions increase in the low fertility scenario briefly, due to the increase in the share of the working age population. By 2100 C.E., there is a substantial decline in relative emissions, leaving our key results unchanged.

## 4. Discussion

The trade-off between economic and environmental priorities is central to the most commonly discussed policies aimed at combating global climate change [4]. It is important to note that population policies can have a positive effect on economic outcomes before considering the feedback from environmental to economic damage. This is the crucial difference with integrated assessment models—which often translate all damages into economic units—that show a positive effect of climate policies on economic outcomes [28, 29]. These feedback benefits would certainly still occur as a result of population policies, but they are not necessary to achieve positive economic outcomes.

While our primary goal is simply to demonstrate that lower population can simultaneously increase income per capita and lower carbon emissions, our results also have substantial implications for policy. First, implementing population-based policies in developing countries could help overcome problems of international burden sharing in the mitigation of climate change [10, 30]. This is especially relevant given high predicted fertility in developing countries and evidence for a high unmet demand for contraceptives [27, 31]. Indeed, under certain burden-sharing agreements, poor African countries are not expected to substantially contribute to emission reductions over the next several decades [30, 32]. Yet, our analysis suggests that moving to a feasible fertility scenario in Nigeria could lower relative emissions by 10% in 2055 and 35% by 2100. Second, since such policies do not have inherent economic trade-offs, they do not suffer from free-rider problems, implying that it may be easier to reach agreements to lower emissions through population-based policies [13,14].

We do not argue that population policies are a panacea for solving environmental and economic problems. In particular, we have not shown that population policies are sufficient to meet reasonable emissions targets on a global scale or even that feasible reductions in fertility would bring emissions below their current level, which would require a reduction in the level of population. Instead, our results suggest that population policies could be a component of the international approach to climate policy. Indeed, given the fact that many countries—especially wealthier countries, China, and Russia—contribute substantially to global carbon emissions despite having low rates of population growth, it is highly unlikely that population policies will be the primary driver of emission reductions. Still, any global emission reductions that are achieved via population policies may not be subject to the economic trade-off central to most other policies and may be easier to implement given the lack of free-rider concerns. To understand what role reduced fertility can play in the reduction of total global carbon emissions, future work would need to extend the analysis presented here to the entire world.

Our analysis has examined the effects of an exogenously lower path of fertility given by the UN projections, rather than the outcome of a specific policy or set of policies. There are many policies that may lead to lower fertility, the most obvious of which is the provision of contraceptives. A number of other policies, however, would also alter fertility in developing countries. As with all decisions, parents have limited resources to allocate to raising children, and as a result, many economic policies will influence fertility rates. In particular, parents must decide how to allocate resources between having more children and investing in the future of each child [33, 34]. There is considerable evidence for this ‘quantity-quality trade-off in the economics literature [35–37]. Thus, policies that increase incentives for investment in education could also lead to lower fertility. Any policy that affects fertility will likely affect the evolution of population, age structure, and income per capita through other avenues, such as the effects of increased taxes or changes in government budgets. Examining the effects of particular policies represents an important area for future research to build on the analysis presented here.

While this analysis has demonstrated the potential for reductions in fertility to simultaneously achieve environmental and economic policy priorities, many opportunities remain to extend the analysis, as noted above. First, the model employed here does not include a detailed representation of the energy sector. Understanding how population, age structure, and income per capita differentially affect the energy intensity ofoutput and carbon intensity ofenergy is an important step towards understanding the mechanisms underlying these results and determining how to design targeted policies that can overcome trade-offs central to most efforts at combating global climate change. Including such mechanisms in the modeling stage of an analysis like ours could also sharpen the quantitative estimates. Second, expanding the geographic scope of the analysis is necessary to more fully understand the role that population policies can play in mitigating global climate change. Finally, evaluation of any particular policy necessitates extending the analysis to include specific reasons for the decline in fertility, rather than taking such a change as exogenous.

## 5. Conclusion

We have demonstrated that lower fertility can simultaneously achieve environmental and economic policy priorities. This stands in stark contrast to most policy options aimed at mitigating global climate change, which involve significant trade-offs between wealth and environmental protection, at least before considering the economic damages caused by reduced environmental quality. Thus, our research suggests that population policies could be part of the global policy response to climate change. Indeed, such policies may receive increased political support because they may not suffer from free-rider problems. We hope that our analysis will spur further research regarding the ability of population polices to combat climate change.

## Supplementary Material

appendix

## Figures and Tables

**Table 1.** Determinants of carbon emissions: GDP per capita and population.

	(1)	(2)	(3)	(4)
Ln pop. (a)	1.364***	1.469***	1.406***	1.439***
	(0.172)	(0.176)	(0.175)	(0.203)
Ln gdppc (b)	0.203***	0.207***	0.206***	0.226***
	(0.042)	(0.044)	(0.044)	(0.052)
% Age 15–64		0.016**	0.016**	0.016**
		(0.007)	(0.007)	(0.007)
% Urban			0.008*	0.014***
			(0.004)	(0.005)
Trade (% of GDP)				0.0002
				(0.0002)
Year FE	Yes	Yes	Yes	Yes

Observations	7133	6426	6426	5679
Countries	156	153	153	147
*R*-squared	0.05	0.05	0.05	0.05
Within *R*-squared	0.02	0.02	0.02	0.02
*P*-value: *a* = *b*	0.000	0.000	0.000	0.000
*P*-value: *a* = 1	0.036	0.009	0.022	0.032

Notes

* *p* < 0.1

** *p* < 0.05

*** *p* < 0.01.

Robust standard errors clustered at country level in parentheses. Equation estimated in first differences. In all specifications, the dependent variable is the natural log of total CO2 emissions. The sample covers 1950–2010. *Within R-squared* is the percentage of variation in the dependent variable explained by the independent variables after removing variation due to time and year fixed effects.

## References

[1] Raupach MR, Marland G, Ciais P, Le Quéré C, Canadell JG, Klepper G and Field CB 2007 Global and regional drivers of acceleratingCO2 emissions Proc. Natl Acad. Sci 104 10288–93 17519334 10.1073/pnas.0700609104 PMC1876160

[2] O’Neill BC, Liddle B, Jiang L, Smith KR, Pachauri S, Dalton M and Fuchs R 2012 Demographic change and carbon dioxide emissions Lancet 380 157–64 22784534 10.1016/S0140-6736(12)60958-1

[3] O’Neill BC, Dalton M, Fuchs R, Jiang L, Pachauri S and Zigova K2010 Global demographic trends and future carbon emissions Proc. Natl Acad. Sci 107 17521–6 20937861 10.1073/pnas.1004581107 PMC2955139

[4] Nordhaus WD 2014 A Question of Balance: Weighing the Options on Global Warming Policies (New Haven, CT: Yale University Press)

[5] Dietz T and Rosa EA 1997 Effects of population and affluence on CO2 emissions Proc. Natl Acad. Sci. 94 175–9 8990181 10.1073/pnas.94.1.175 PMC19273

[6] York R, Rosa EA and Dietz T 2003 STIRPAT, IPAT and IPACT: analytic tools for unpacking the driving forces of environmental impacts Ecolog. Econ 46 351–65

[7] Liddle B 2015 What are the carbon emissions elasticities for income and population? bridging STIRPAT and EKC via robust heterogeneous panel estimates Glob. Environ. Change 31 62–73

[8] Alcott B 2010 Impact caps: why population, affluence and technology strategies should be abandoned J. Cleaner Prod 18 552–60

[9] AshrafQ H, Weil DN and Wilde J 2013 The effect of fertility reduction on economic growth Population Dev. Rev 39 97–130 10.1111/j.1728-4457.2013.00575.x PMC4267474 25525283

[10] Brunnée J and Streck C 2013 The UNFCCC as a negotiation forum: towards common but more differentiated responsibilities Clim. Policy 13 589–607

[11] United Nations (2015). Adoption of The Paris Agreement.

[12] Aldy JE 2012 Climate negotiators create an opportunity for scholars Science 337 1043–4 22936761 10.1126/science.1223836

[13] Stavins RN 2011 The problem of the commons: still unsettled after 100years Am. Econ. Rev 101 81–108

[14] Nordhaus W 2015 Climate clubs: overcoming free-riding in international climate policy Am. Econ. Rev 105 1339–70

[15] Liddle B 2014 Impact of population, age structure, and urbanization on carbon emissions/energy consumption: evidence from macro-level, cross-country analyses Population Environ. 35 286–304

[16] Dalton M, O’Neill B, Prskawetz A, Jiang L and Pitkin J 2008 Population aging and future carbon emissions in the united states Energy Econ. 30 642–75

[17] Holdren JP and Ehrlich PR 1974 Human population and the global environment: population growth, rising per capita material consumption, and disruptive technologies have made civilization a global ecological force Am. Sci 62 282–92 4832978

[18] Chertow MR 2000 The IPAT equation and its variants J. Ind. Ecol 4 13–29

[19] Jorgenson AK and Clark B 2010 Assessing the temporal stability of the population / environment relationship in comparative perspective: a cross-national panel study of carbon dioxide emissions, 1960–2005 Population Environ. 32 27–41

[20] Liddle B 2013 Consumption-driven environmental impact and age structure change in OECD countries: a cointegration-stirpat analysis Demogr. Res 24 749–70

[21] Boden TA, Marland G and Andres RJ 2015 Global, Regional, and National Fossil-Fuel CO2 Emissions vol 10(Carbon Dioxide Information Analysis Center, Oak Ridge National Laboratory, US Department of Energy, Oak Ridge, TN, USA)

[22] Feenstra RC, Inklaar R and Timmer MP 2015 The next generation of the Penn World Table Am. Econ. Rev 105 3150–82

[23] Wagner M 2008 The carbon Kuznets curve: a cloudy picture emitted by bad econometrics? Resour. Energy Econ 30 388–408

[24] Jorgenson AK and Clark B 2013 The relationship between national-level carbon dioxide emissions and population size: an assessment of regional and temporal variation, 1960–2005 PLoSOne 8 e57107 10.1371/journal.pone.0057107 PMC3577780 23437323

[25] Knight KW, Rosa EA and Schor JB 2013 Could working less reduce pressures on the environment? a cross-national panel analysis of OECD countries, 1970–2007 Glob. Environ. Change 23 691–700

[26] Liddle B and Lung S 2010 Age-structure, urbanization, and climate change in developed countries: revisiting stirpat for disaggregated population and consumption-related environmental impacts Population Environ. 31 317–43

[27] United Nations, Department of Economic and Social Affairs, Population Divison (2015). World Population Prospects: The 2015 Revision, DVD Edition.

[28] Nordhaus WD 2013 The Climate Casino: Risk, Uncertainty, and Economics for a Warming World (New Haven, CT: Yale University Press)

[29] Golosov M, Hassler J, Krusell P and Tsyvinski A 2014 Optimal taxes on fossil fuel in general equilibrium Econometrica 82 41–88

[30] Cazorla M and Toman M International equity and climate change policy Climate Change Economics and Policy: An RFF Anthology vol 235 (Washington, DC: Resource For the Future)

[31] Gillespie D, Ahmed S, Tsui A and Radloff S 2007 Unwanted fertility among the poor: an inequity? Bull. World Health Org 85 100–7 17308730 10.2471/BLT.06.033829 PMC2636279

[32] Nordhaus WD 2010 Economic aspects of global warming in a post-C open hagen environment Proc. Na tl Acad. Sci 107 11721–6 10.1073/pnas.1005985107 PMC2900661 20547856

[33] Becker GS 1960 An Economic Analysis of Fertility (NBER) pp 209–40

[34] Galor O 2011 Unified Growth Theory (Princeton, NJ: Princeton University Press)

[35] Bleakley H and Lange F 2009 Chronic disease burden and the interaction of education, fertility, and growth Rev. Econ. Stat 91 52–65 24163482 10.1162/rest.91.1.52 PMC3806284

[36] Aaronson D, Lange F and Mazumder B2014 Fertility transitions along the extensive and intensive margins Am. Econ. Rev 104 3701–24

[37] Galor O 2012 The demographic transition: causes and consequences Cliometrica 61–28 10.1007/s11698-011-0062-7 PMC4116081 25089157

