# Supplementary material for: Is faster economic growth compatible with reductions in carbon emissions? The role of diminished population growth
Source: Environ Res Lett. Author manuscript; Available in PMC 2020 Feb 12. (PMC7015536; doi:10.1088/1748-9326/12/1/014003)
Supplement: appendix [file NIHMS1013206-supplement-appendix.pdf]

Appendix to: Is economic growth compatible with reductions in carbon emissions? Investigating the impacts of diminished population growth

Gregory Casey and Oded Galor

1 Supplemental Information

1.1 Data

Our emissions data comes from Oak Ridge National Laboratory and are standard in the literature [1]. The dependent variable is *Total* CO_2 *Emissions from Fossil Fuels*, which is measured in thousands of metric tons of Carbon. The estimates of carbon emissions are constructed using fossil fuel inputs in production [1, 2].

We take output and population data from version 8.0 of the Penn World Tables [3, 4]. The Penn World Tables measure real GDP, which accounts for differences in prices across countries. A major innovation of version 8.0 is that there are now several measures of real GDP. For our analysis, we use output-side real GDP, *rgdpo*, which measures the level of production, as opposed to consumption, in the economy. This is best choice because our measure of CO_2 is calculated based on fossil fuel production, rather than consumption. GDP is measured in 2005 USD. We also take population values, *pop*, from the Penn World Tables. Output per capita is just the ratio of the two variables from the PWT. The data cover 1950-2010. We use alternate measures of income in robustness exercises.

We take data on the population age structure, urbanization rates, and trade from the World Bank's World Development Indicators (WDI) database. Trade is measured as: $(exports+imports)/GDP$. We also use the exchange-rate adjusted measure of income per capita from the WDI in robustness exercises.

We drop any country from the analysis that has GDP per capita greater than 100,000 USD 2005 in any year. We take these high GDP numbers to indicate that true production levels are not well measured by the PWT approach. This eliminates Bermuda, Brunei, Kuwait, Qatar, and Saudi Arabia. There are also two country-year observations with negative emissions, Senegal in 1968 and Yemen in 1990, which we drop from the analysis. Our qualitative findings are unchanged if we include all of these observations.

We also remove Israel, Cyprus, and Malta, which are clear outliers that bias the results in favor of finding much larger coefficients on population.

1.2 Regression Analysis

Regressions were performed in *Stata* statistical software. *Within R-squared* is calculated using the user-created module *ivreg2* [5].

1.3 Demographic Simulation

Ashraf *et al.* (2014) construct an economic-demographic simulation model that uses standard economic modeling to predict the aggregate effects of an exogenous reduction in fertility [6]. They study Nigeria from 2005-2100 under the medium and low fertility projections from the United Nations [7]. The output of the model is future paths of population, age distribution, and output per capita under different fertility scenarios and parameters estimates. We employ the results from their main exercise, which has zero technology growth and the authors' preferred estimates for each of the key parameters. We update the analysis to use the newest version of the UN projections [8]. We then combine the model's projections with our econometric estimates to construct predictions for relative carbon emissions under the two scenarios using the following equation:

$$\begin{aligned}\frac{emissions_{j,t}}{emissions_{k,t}} = exp\Bigg(&0.226 * \big(ln(gdppc_{j,t}) - ln(gdppc_{k,t})\big) \\ &+ 1.439 * \big(ln(pop_{j,t}) - ln(pop_{k,t})\big), \\ &+ 0.016 * \big(ln(WA_{j,t}) - ln(WA_{k,t})\big)\Bigg),\end{aligned} \tag{1}$$

where j denotes outcomes under the low fertility scenario, k denotes outcomes under the medium fertility scenario, and WA is the percent of the population between ages 15-65.

We make a few modifications to the starting values in the model. The original model does not impose the actual levels of GDP for Nigeria and, instead, is only concerned with the ratio between the two scenarios. We impose the level of GDP and physical capital in 2005 using the data from PWT version 8.0 for Nigeria in 2005. Consistent with the regression data, we use output side real GDP, *rgdpo*, which is 220,303.3 million 2005 USD. For the capital stock, we use *rkna*, which is 'Capital Stock at Constant National Prices', yielding 339,150.3 million 2005 USD. Unfortunately, the capital stock is not available as an output-side measure. We then normalize the 'Fixed Stock of Land' to 1. When combined with the 2005 stock of human capital, which is already calculated in the original model, this yields a level of technology of $A = 4.46$.

Our adjustments have a very slight effect on the ratio of output per capita that comes from the the model. Specifically, in 2100, the original formulation leads output per capita that is 15.80% higher than in the low fertility scenario. With our adjustments, the output per capita ratio in 2100 is 15.86% higher in the low fertility scenario. The ratio of population and the fraction of the population of working age are exogenous and unaffected by imposing the initial level of GDP.

2 Summary Statistics

Table A1: Summary Statistics for Unbalanced Sample 1950-2010

	mean	sd	min	max
CO_2 Emissions (thous. metric tons of C)	38,406	153,483	5	2,259,856
GDP per Capita (2005 USD)	8,300	9,478	163	59,640
Pop. (millions)	37.67	128.4	0.06	1,318
% Age 15-64	58.59	6.74	45.92	75.18
% Urban	48.84	24.13	2.19	100.0
Trade (% of GDP)	73.04	51.71	0.02	531.7
Observations	5679			
Countries	147			

3 Results from Levels Regression

Table A2: Determinants of Carbon Emissions: Levels Regression

	(1)	(2)	(3)	(4)	(5)
Ln pop. (a)	1.877***	1.654***	1.781***	1.685***	1.585***
	(0.179)	(0.209)	(0.212)	(0.224)	(0.248)
Ln gdppc (b)	0.697***	0.650***	0.589***	0.576***	0.515***
	(0.098)	(0.097)	(0.111)	(0.114)	(0.121)
Ln gdppc squared (c)		-0.076**	-0.060*	-0.056*	-0.068*
		(0.033)	(0.032)	(0.032)	(0.035)
% Age 15-64			0.024**	0.023**	0.024**
			(0.009)	(0.009)	(0.009)
% Urban				0.009*	0.010**
				(0.005)	(0.005)
Trade (% of GDP)					0.0006
					(0.0006)
Country FE	Yes	Yes	Yes	Yes	Yes
Year FE	Yes	Yes	Yes	Yes	Yes
Observations	7291	7291	6581	6581	5840
Countries	156	156	153	153	147
R-Squared	0.98	0.98	0.98	0.98	0.98
Within R-Squared	0.328	0.339	0.336	0.342	0.361
P-value: a = b	0.000	0.000	0.000	0.000	0.000
P-value: a = 1	0.000	0.002	0.000	0.003	0.019

Notes: $*p < 0.1, **p < 0.05, ***p < 0.01$. Robust standard errors clustered at country level in parentheses. Equation estimated in first differences. In all specifications, the dependent variable is the natural log of total CO_2 emissions. The sample covers 1950-2010. *Within R-squared* is the percentage of variation in the dependent variable explained by the independent variables after removing variation due to time and year fixed effects.

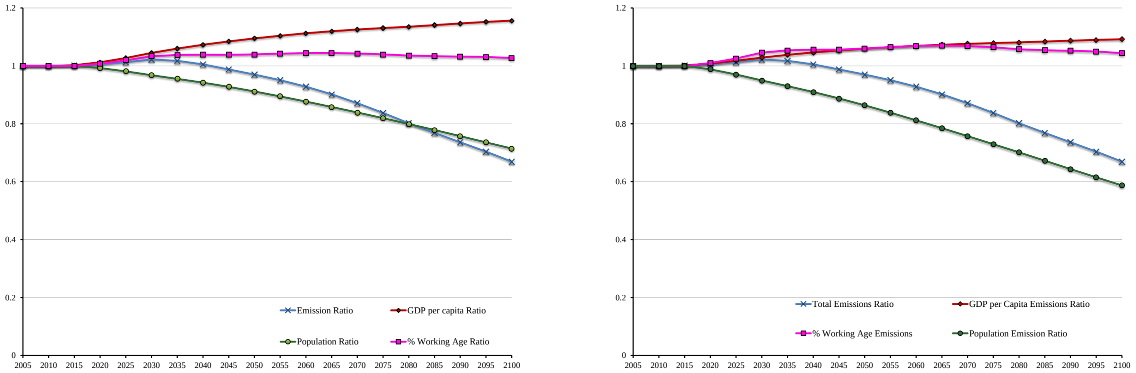

Figure A1: Results from the economic-demographic model. All variables are the the ratio of the outcome of the low fertility scenario over the medium fertility scenario. *Panel A* (left) plots the main outcome variables. *Panel B* (right) decomposes the difference in emissions between sources.

4 Results from Balanced Sample

Table A3: Determinants of Carbon Emissions: Balanced Sample

	(1)	(2)	(3)	(4)
Ln pop. (a)	1.160***	1.197***	1.165***	1.127***
	(0.217)	(0.210)	(0.210)	(0.221)
Ln gdppc (b)	0.206***	0.196***	0.195***	0.214***
	(0.050)	(0.051)	(0.050)	(0.051)
% Age 15-64		0.032***	0.032***	0.035***
		(0.006)	(0.006)	(0.006)
% Urban			0.005	0.005
			(0.006)	(0.006)
Trade (% of GDP)				0.0005
				(0.0005)
Year FE	Yes	Yes	Yes	Yes
Observations	2940	2450	2450	2332
Countries	49	49	49	48
R-Squared	0.09	0.10	0.10	0.11
Within R-Squared	0.022	0.030	0.030	0.037
P-value: a = b	0.000	0.000	0.000	0.000
P-value: a = 1	0.464	0.353	0.435	0.568

Notes: $*p < 0.1, **p < 0.05, ***p < 0.01$. Robust standard errors clustered at country level in parentheses. Equation estimated in first differences. In all specifications, the dependent variable is the natural log of total CO_2 emissions. The sample covers 1950-2010. *Within R-squared* is the percentage of variation in the dependent variable explained by the independent variables after removing variation due to time and year fixed effects.

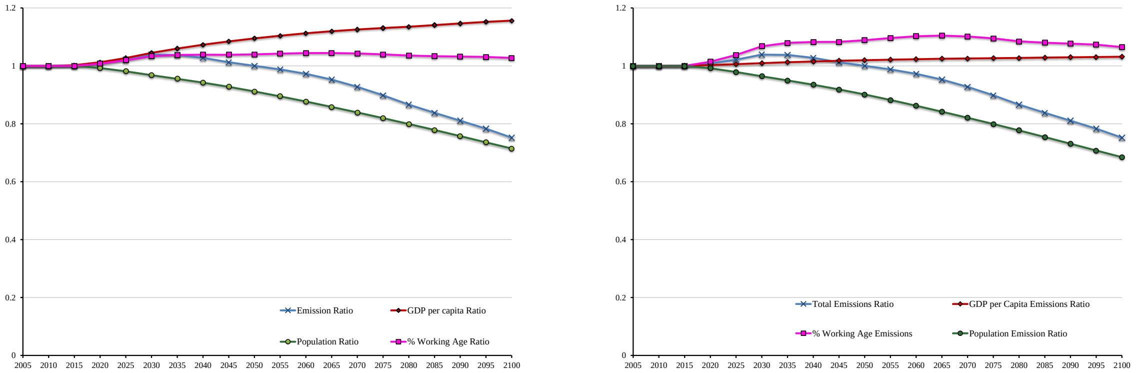

Figure A2: Results from the economic-demographic model. All variables are the the ratio of the outcome of the low fertility scenario over the medium fertility scenario. *Panel A* (left) plots the main outcome variables. *Panel B* (right) decomposes the difference in emissions between sources.

5 Results with Total GDP

Table A4: Determinants of Carbon Emissions: Total GDP

	(1)	(2)	(3)	(4)
Ln pop. (a)	1.161***	1.262***	1.200***	1.213***
	(0.169)	(0.179)	(0.177)	(0.199)
Ln gdp (b)	0.203***	0.207***	0.206***	0.226***
	(0.042)	(0.044)	(0.044)	(0.052)
% Age 15-64		0.016**	0.016**	0.016**
		(0.007)	(0.007)	(0.007)
% Urban			0.008*	0.014***
			(0.004)	(0.005)
Trade (% of GDP)				0.0002
				(0.0002)
Year FE	Yes	Yes	Yes	Yes
Observations	7133	6426	6426	5679
Countries	156	153	153	147
R-Squared	0.05	0.05	0.05	0.05
Within R-Squared	0.017	0.020	0.020	0.023
P-value: a = b	0.000	0.000	0.000	0.000
P-value: a = 1	0.342	0.145	0.260	0.285

Notes: $*p < 0.1, **p < 0.05, ***p < 0.01$. Robust standard errors clustered at country level in parentheses. Equation estimated in first differences. In all specifications, the dependent variable is the natural log of total CO_2 emissions. The sample covers 1950-2010. *Within R-squared* is the percentage of variation in the dependent variable explained by the independent variables after removing variation due to time and year fixed effects.

6 Results with Consumption Side Measure of GDP

Table A5: Determinants of Carbon Emissions: Consumption-side

	(1)	(2)	(3)	(4)
Ln pop. (a)	1.370***	1.459***	1.405***	1.449***
	(0.166)	(0.170)	(0.168)	(0.199)
Ln gdppc (cons. side) (b)	0.302***	0.302***	0.300***	0.324***
	(0.056)	(0.060)	(0.060)	(0.073)
% Age 15-64		0.014*	0.013*	0.013*
		(0.007)	(0.007)	(0.007)
% Urban			0.007*	0.011**
			(0.004)	(0.005)
Trade (% of GDP)				0.0003
				(0.0002)
Year FE	Yes	Yes	Yes	Yes
Observations	7133	6426	6426	5679
Countries	156	153	153	147
R-Squared	0.06	0.06	0.06	0.06
Within R-Squared	0.026	0.028	0.028	0.031
P-value: a = b	0.000	0.000	0.000	0.000
P-value: a = 1	0.027	0.008	0.018	0.025

Notes: $*p < 0.1, **p < 0.05, ***p < 0.01$. Robust standard errors clustered at country level in parentheses. Equation estimated in first differences. In all specifications, the dependent variable is the natural log of total CO_2 emissions. The sample covers 1950-2010. *Within R-squared* is the percentage of variation in the dependent variable explained by the independent variables after removing variation due to time and year fixed effects.

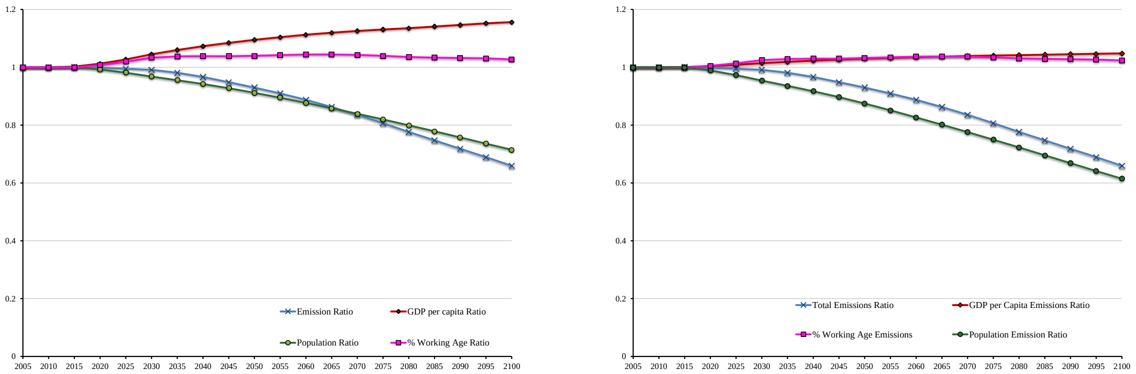

Figure A3: Results from the economic-demographic model. All variables are the the ratio of the outcome of the low fertility scenario over the medium fertility scenario. *Panel A* (left) plots the main outcome variables. *Panel B* (right) decomposes the difference in emissions between sources.

7 Results with National Accounts Measure of GDP

Table A6: Determinants of Carbon Emissions: National Accounts

	(1)	(2)	(3)	(4)
Ln pop. (a)	1.437***	1.517***	1.467***	1.566***
	(0.174)	(0.178)	(0.174)	(0.211)
Ln gdppc (nat'l acct.) (b)	0.500***	0.512***	0.509***	0.609***
	(0.077)	(0.084)	(0.084)	(0.095)
% Age 15-64		0.010	0.010	0.008
		(0.007)	(0.006)	(0.006)
% Urban			0.006*	0.009*
			(0.004)	(0.005)
Trade (% of GDP)				0.0002
				(0.0002)
Year FE	Yes	Yes	Yes	Yes
Observations	7133	6426	6426	5679
Countries	156	153	153	147
R-Squared	0.06	0.07	0.07	0.07
Within R-Squared	0.035	0.038	0.038	0.045
P-value: a = b	0.000	0.000	0.000	0.000
P-value: a = 1	0.013	0.004	0.008	0.008

Notes: $*p < 0.1, **p < 0.05, ***p < 0.01$. Robust standard errors clustered at country level in parentheses. Equation estimated in first differences. In all specifications, the dependent variable is the natural log of total CO_2 emissions. The sample covers 1950-2010. *Within R-squared* is the percentage of variation in the dependent variable explained by the independent variables after removing variation due to time and year fixed effects.

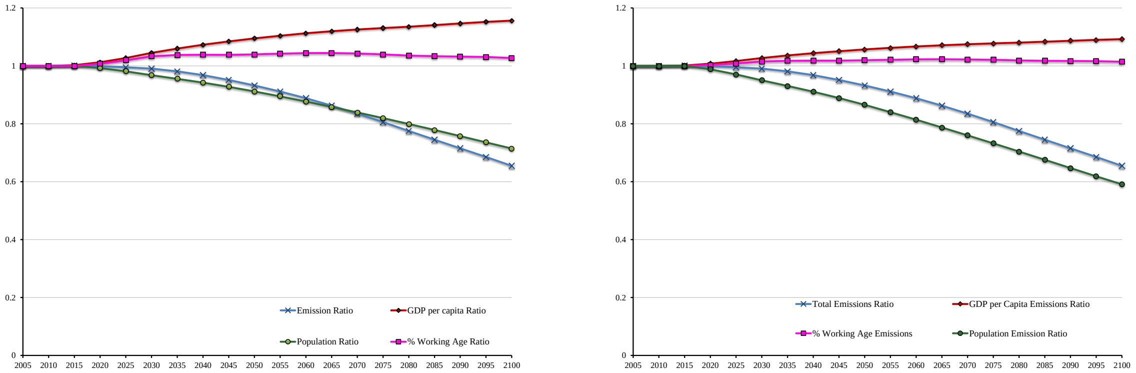

Figure A4: Results from the economic-demographic model. All variables are the ratio of the outcome of the low fertility scenario over the medium fertility scenario. *Panel A* (left) plots the main outcome variables. *Panel B* (right) decomposes the difference in emissions between sources.

8 Results with WDI Measure of GDP

Table A7: Determinants of Carbon Emissions: WDI

	(1)	(2)	(3)	(4)
Ln pop. (a)	1.485***	1.553***	1.496***	1.568***
	(0.221)	(0.216)	(0.214)	(0.226)
Ln gdppc (wdi) (b)	0.547***	0.544***	0.541***	0.588***
	(0.082)	(0.083)	(0.083)	(0.090)
% Age 15-64		0.014**	0.014**	0.011*
		(0.006)	(0.006)	(0.006)
% Urban			0.007	0.010*
			(0.005)	(0.005)
Trade (% of GDP)				0.0004
				(0.0002)
Year FE	Yes	Yes	Yes	Yes
Observations	5817	5755	5755	5549
Countries	153	151	151	146
R-Squared	0.07	0.07	0.07	0.07
Within R-Squared	0.040	0.041	0.042	0.044
P-value: a = b	0.000	0.000	0.000	0.000
P-value: a = 1	0.030	0.012	0.022	0.013

Notes: $*p < 0.1, **p < 0.05, ***p < 0.01$. Robust standard errors clustered at country level in parentheses. Equation estimated in first differences. In all specifications, the dependent variable is the natural log of total CO_2 emissions. The sample covers 1950-2010. *Within R-squared* is the percentage of variation in the dependent variable explained by the independent variables after removing variation due to time and year fixed effects.

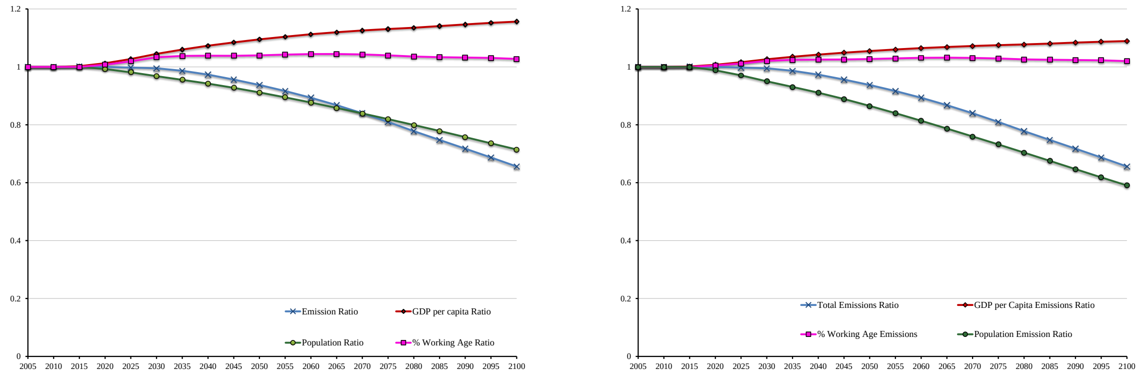

Figure A5: Results from the economic-demographic model. All variables are the the ratio of the outcome of the low fertility scenario over the medium fertility scenario. *Panel A* (left) plots the main outcome variables. *Panel B* (right) decomposes the difference in emissions between sources.

References

[1] T. A. Boden, G. Marland, and R. J. Andres, "Global, regional, and national fossil-fuel CO_2 emissions," *Carbon Dioxide Information Analysis Center, Oak Ridge National Laboratory, US Department of Energy, Oak Ridge, Tenn., USA doi*, vol. 10, 2015.

[2] G. Marland and R. M. Rotty, "Carbon dioxide emissions from fossil fuels: a procedure for estimation and results for 1950–1982," *Tellus B*, vol. 36, no. 4, pp. 232–261, 1984.

[3] R. C. Feenstra, R. Inklaar, and M. P. Timmer, "The next generation of the Penn World Table," *American Economic Review*, vol. 105, no. 10, pp. 3150–82, 2015.

[4] R. C. Feenstra, R. Inklaar, and M. Timmer, *PWT 8.0–a user guide*, 2013.

[5] C. Baum, M. Schaffer, and S. Stillman, "ivreg2: Stata Module for Extended Instrumental Variables/2SLS, GMM and AC/HAC, LIML, and k-class Regression." `http://ideas.repec.org/c/boc/bocode/s425401.html`, 2010.

[6] Q. H. Ashraf, D. N. Weil, and J. Wilde, "The effect of fertility reduction on economic growth," *Population and Development Review*, vol. 39, no. 1, pp. 97–130, 2013.

[7] United Nations, "World Population Prospects: The 2010 Revision," 2010.

[8] United Nations, "World population prospects: The 2015 revision, dvd edition," 2015.
